# Modification of Hemodialysis Membranes for Efficient Circulating Tumor Cell Capture for Cancer Therapy

**DOI:** 10.3390/molecules26164845

**Published:** 2021-08-10

**Authors:** Gabor Jarvas, Dora Szerenyi, Jozsef Tovari, Laszlo Takacs, Andras Guttman

**Affiliations:** 1 Research Institute of Biomolecular and Chemical Engineering, Faculty of Engineering, University of Pannonia, 8200 Veszprem, Hungary; gabor.jarvas@captecmedical.com (G.J.); szerenyi.dora@mukki.richem.hu (D.S.); 2 CAPTEC Medical Ltd., 1124 Budapest, Hungary; 3 Department of Experimental Pharmacology, National Institute of Oncology, 1122 Budapest, Hungary; tozsi@oncol.hu; 4 Laboratory of Monoclonal Antibody Proteomics, Department of Human Genetics, Faculty of Medicine, University of Debrecen, 4032 Debrecen, Hungary; laszlo.takacs@biosys-intl.com

**Keywords:** hemodialysis, circulating tumor cell, capture, therapy

## Abstract

Background: It is well known that more than 90% of cancer deaths are due to metastases. However, the entire tumorigenesis process is not fully understood, and it is evident that cells spreading from the primary tumor play a key role in initiating the metastatic process. Tumor proliferation and invasion also elevate the concentration of regular and irregular metabolites in the serum, which may alter the normal function of the entire human homeostasis and possibly causes cancer metabolism syndrome, also referred to as cachexia. Methods: We report on the modification of commercially available hemodialysis membranes to selectively capture circulating tumor cells from the blood stream by means of immobilized human anti-EpCAM antibodies on the inner surface of the fibers. All critical steps are described that required in situ addition of the immuno-affinity feature to hemodialyzer cartridges in order to capture EpCAM positive circulating tumor cells, which represents ~80% of cancer cell types. Results: The cell capture efficiency of the suggested technology was demonstrated by spiking HCT116 cancer cells both into buffer solution and whole blood and run through on the modified cartridge. Flow cytometry was used to quantitatively evaluate the cell clearance performance of the approach. Conclusions: The suggested modification has no significant effect on the porous structure of the hemodialysis membranes; it keeps its cytokine removal capability, addressing cachexia simultaneously with CTC removal.

## 1. Introduction

Cancer is the second leading cause of mortality globally and was responsible for ~10 million deaths in 2020 [1], more than 90% of which were due to metastases [2,3]. While the entire tumorigenesis process is not fully understood, it is evident that cells spreading from the primary solid tumor play a key role in initiating the metastatic process [4,5,6,7,8,9]. Such detached cells are referred to as circulating tumor cells (CTCs) and are often utilized in tumor prognosis estimation, cancer diagnosis, treatment monitoring and decision making [4,10,11,12]. Currently, the clinical potency of CTC capture is exploited in liquid biopsy as a good alternative to surgical tissue sampling [13,14,15,16,17]. Although much progress has been made in CTC enumeration and isolation during the last decades, only two systems obtained FDA approval by late 2020 [18]. The most challenging aspects of CTC isolation are their low copy number and heterogeneity [19]. On the other hand, for the time being, therapeutic utilization of CTC removal is rather limited to methods that may be applicable in vivo by directly neutralizing CTCs using nanomaterials such as liposomes and gold nanoparticles [20].

The utilization of microfluidic devices has significantly increased during the last decade, but their limited throughput (up to 20 mL/h, [14]) still restrains their utilization in high yield CTC removal. The most promising avenue to exploit the direct therapeutic potential of CTC removal is their elimination from the bloodstream by extracorporeal procedure (i.e., similar to hemodialysis) in order to slow down or even prevent the metastatic process. Others proposed extracorporeal photoimmunotherapy to eliminate CTCs from the circulation [21]. Unfortunately, the method was barely effective due to the high light-absorbance of red blood cells [22], and its technical realization was rather complex for potential cancer therapeutic application but proved somewhat curative to treat leukemia [23,24]. Edelman et al. investigated the use of a leukocyte depletion filter to remove tumor cells derived from urologic malignancies [25]. The authors suggested their method for intraoperative autotransfusion during uro-oncologic surgery. A similar approach was reported by Perseghin et al. for leukocyte filtration of intraoperative blood salvage to reduce the risk of tumor cell transmission. Such technologies have reportedly common drawbacks [26] such as: (1) they cannot be utilized in pre/post operation and (2) their removal mechanism is not CTC specific. In 2015, Gaitas and Kim published a pioneering paper on high volume removal and collection of CTCs by immobilized human anti-EpCAM antibodies [27]. They used chemically modified ordinary plastic with immobilized antibodies in the interior surface to achieve a promising CTC capture rate, both from spiked media and whole blood; however, their technology does not address cytokine removal.

Mammalian cancer development starts as a localized, uncontrolled cell growth that subsequently progresses to a systemic disease. This systemic syndrome, termed cancer-associated cachexia (CAC), is a major cause of cancer morbidity and mortality [28]. CAC is an extremely complex metabolic disorder often resulting in multiorgan dysfunction. Interferon gamma, interleukin-1, interleukin-6, and tumor necrosis factor-alpha are the most frequently associated cytokines associated with cachexia pathogenesis [29], but many others have important roles as well [30]. Removal or neutralization of such cytokines is proved to be beneficial in attenuating the progression of various diseases, such as sepsis and cancer [31,32,33,34,35].

In this study, we report on the in situ modification technology of commercially available hemodialysis membranes to selectively capture circulating tumor cells from the blood stream by means of immobilized anti-human EpCAM antibodies at the interior surface of the fibers. In addition to demonstrating the feasibility of the suggested technology for efficient CTC capture, we paid special attention to keep the filtering capability of the membrane unaffected, thus capable of simultaneously addressing CAC.

## 2. Results and Discussion

**Figure 1:**
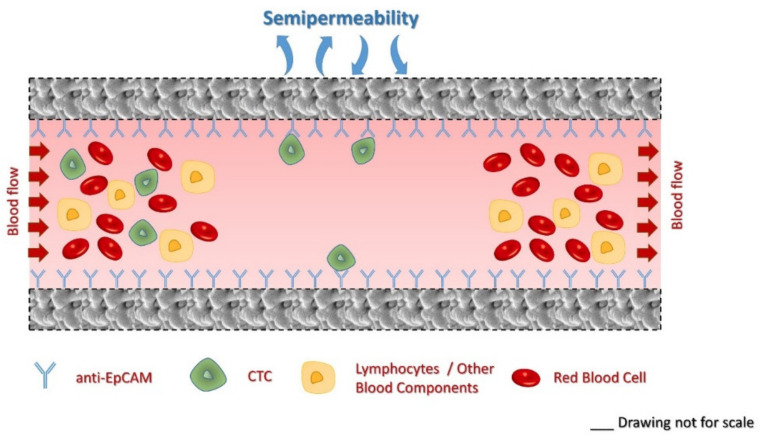
Schematic representation of the simultaneous CTC capture and hemodialysis processes.

In this study, we report on the in situ modification of commercially available hemodialysis membranes to selectively capture circulating tumor cells by immobilized anti-human EpCAM antibodies while maintaining its hemodialysis capability. Capture efficiency from buffer and mice blood was investigated by flow cytometry. The filtering capability of the membranes after the suggested modification was also examined. The scheme of the parallel CTC capture and hemodialysis is shown in Figure 1.

**Figure 2:**
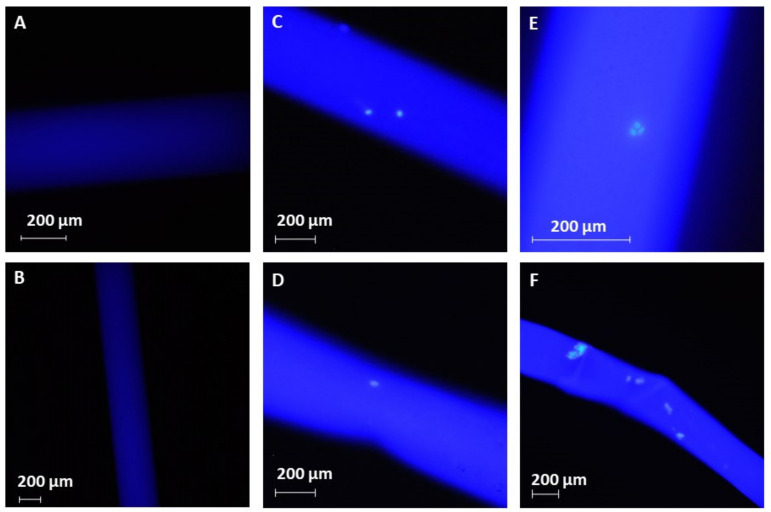
Fluorescence images of the treated fibers. Panels (**A**,**B**): background illumination of the untreated fibers. Panels (**C**–**F**): efficient HCT116 cell capture (green signal) from spiked PBS buffer. The intensive blue illumination in panels (**C**–**F**) demonstrates the efficient anti-EpCAM immobilization. Images were taken using a Nikon Eclipse Ni fluorescence microscope equipped with a DAPI (ex.: 375 nm/em.: 460) bandpass filter cube.

Successful immobilization of the anti-EpCAM molecules was confirmed by fluorescent microscopy. The FITC labeled anti-EpCAM molecules were efficiently immobilized onto the polysulfone membranes, as demonstrated by the homogenous illumination in Figure 2C–F in the fluorescent microscope images shown in Figure 2. The uniform illumination pattern verified the efficiency of the suggested run-through immobilization approach, which ensured effective surface coverage, even under in situ conditions. Based on preliminary fluorimetry determination, it is estimated that ~0.7 ng/mm^2^ (5 fmol/mm^2^) anti-EpCAM was immobilized on the surface of the fibers. Figure 2 also demonstrates the feasibility of the suggested technology, as the captured cells are clearly visible in Figure 2C–F, while Figure 2A,B show the autofluorescence of the untreated hollow fibers.

**Figure 3:**
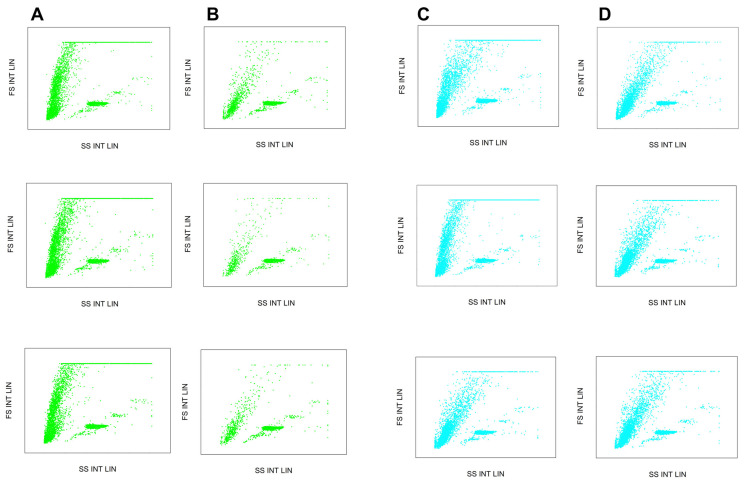
Quantitative flow cytometry assessment of CTC capture efficiency. Dot plots show the populations of HCT116 cells as well as the applied internal standard. Columns (**A**,**B**): initial and run-through events of the EpCAM immobilized fiber experiments. Rows show the repeatability of the triplicate measurements. Columns (**C**,**D**): initial and run through events of the control fiber experiments.

The capture efficiency was quantitatively monitored by flow cytometry (Figure 3) by evaluating the spiked buffer and total blood samples. In both instances, the cell count of the initial and run-through cell suspension was considered. From the spiked PBS buffer, an average of 84% of the initial cell count was captured using the anti-EpCAM activated fibers, while the control system non-specifically captured an average of 35%. The difference in the capture efficiency of the active and control setups indicated the actual performance of the active fibers, i.e., ~2.6 × 10^12^ cells/m^2^ membrane (Table 1). The relatively high cell capture capability of the control system was probably due to the lack of blocking the non-specific binding sites. Cell capture efficiency can be further improved by applying higher anti-EpCAM concentration of the coupling buffer as well as prolonging the contact times. Non-specific binding can be reduced by adsorption of serum albumin addition or low molecular weight polyethylene glycol to the membrane surface [36]. Alternatively, the non-specific binding sites can be blocked by priming the prepared dialyzer using the patient’s blood, similar to how it is routinely applied during the standard hemodialysis procedure and executed before each treatment accordingly [37,38].

Total mice blood was utilized to determine the capture efficiency in a complex in vitro model system. In this case, an average of 69% of the initially loaded cells was captured using the anti-EpCAM activated fibers, while the control system only captured about 21%. The approximately 15% less capture efficiency with this approach was probably due to the heparinization during blood collection, as suggested by others [27], i.e., heparin may non-specifically bind to the antibodies [39] or specifically interact with their glycocalyx [39], decreasing binding efficiency. Under these circumstances, the actual performance of the EpCAM activated fibers was found to be as high as ~2.7 × 10^12^ cells/m^2^ membrane (Table 1). Please note, the reported cell count values were not subjected to any statistical analysis as the current study focused on the exploration of the feasibility of the suggested method rather than providing technological development details.

As a first approximation, we consider that the obtained high capture efficiency was due to the very high initial cell count introduced onto the column. On the other hand, the high initial concentration was necessary to ensure reliable precision of the flow cytometry measurements. Comparing the utilized initial cell concentration to a realistic value of 5 CTCs/7.5 mL blood (i.e., the threshold to distinguish patients with short versus long-progression free survival [40,41]) and assuming a linear relationship between the initial cell concentration and the capturing efficiency, ~2.1 × 10^6^ cells/m^2^ membrane EpCAM positive cell capture capacity is expected with our suggested protocol. An average patient with 80 kg body weight has ~5 L blood, assuming the threshold of 5 CTCs/7.5 mL blood concentration results in ~3000 CTCs, which is significantly lower than that of the capturing ability of our modified EpCAM immobilized dialyzer with more than 1 m^2^ surface area. Comparing the specific capture capacity of the modified dialyzer (10^6^ cells) with the targeted CTCs in the range of up to 10^3^, it is expected that the suggested technology has the ability to capture the great majority of CTCs in a real extracorporeal environment.

The utilization of the anti-EpCAM antibody limits the application domain of the suggested technology as not all cancer types feature EpCAM. High and frequent EpCAM expression has been proved in various epithelial-derived tumors, as well as in breast cancer, colorectal cancer, prostate cancer and lung cancer [17,42,43], which represents the vast majority of carcinomas [44]. Such limitation can be overcome by the application of antibody cocktails containing both specific and general tumor markers. Furthermore, the medical condition of the patients (e.g., comorbidity or vein availability) may limit the application of the suggested technology, which will be decided by the treating medical team.

**Figure 4:**
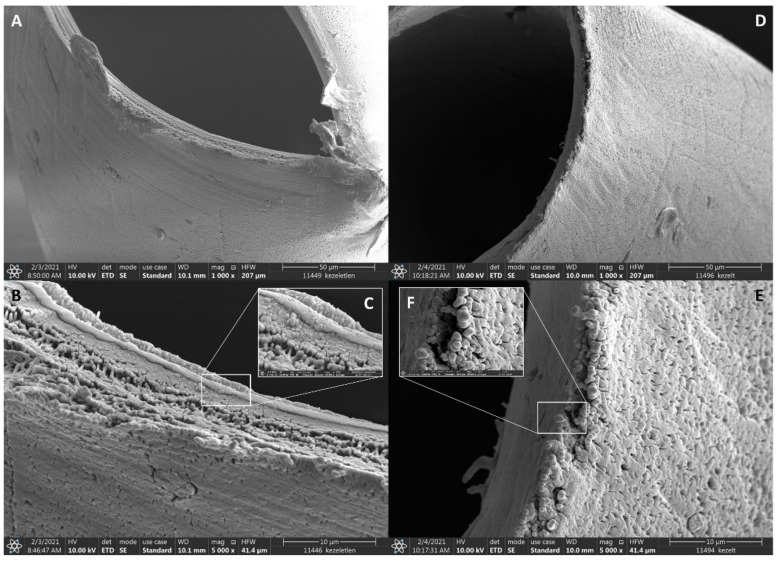
Scanning electron microscope images of the untreated polysulfone membrane (**A**–**C**, negative control) and after anti-EpCAM immobilization (**D**–**F**).

After the capture efficiency studies, special attention was paid to explore any potential changes in the membrane structure possibly caused by the antibody immobilization process. Scanning electron microscope (SEM) imaging was utilized to visualize the membrane microstructure. By comparing the different magnification scale pictures in Figure 4, no significant chemical or physical changes were observed, i.e., after the treatment (Figure 4D–F), both the multilayer structure and the porous surface pattern (pore size, pore size distribution, pore density, pore geometry, and surface roughness) remained very similar to the original membrane (Figure 4A–C). Additionally, SEM pictures verified the absence of any visible residues (e.g., deposition of reagent excess, protein conglomerates, etc.) on the surface of the preserved membrane structure. Absolute water permeability of the untreated and modified dialyzers was also examined to characterize the overall ability of the membranes to remove uremic toxins, inflammatory mediators and cytokines during CTC capture. The resulted absolute permeability values of 42.24 mL/h/mm Hg/m^2^ of the untreated and 39.57 mL/h/mm Hg/m^2^ of the modified membrane clearly demonstrated that the suggested in situ immobilization approach had no significant effect on keeping the dialyzer capability of the membrane. At this stage, no further specific clearance features were investigated in addition to the hydraulic permeability measurements.

## 3. Materials and Methods

### 3.1. Chemicals

Albumin from human serum (HSA), ethanolamine, picoline borane, Hoechts 33342 dye, RPMI 1640 cell culture media, and phosphate-buffered saline (PBS) were purchased from Sigma-Aldrich (St Louis, MO, USA). Fetal bovine serum, fluorescein isothiocyanate (FITC) labeled anti-EpCAM and 1% Penicillin-Streptomycin were from Thermo Fisher Scientific (Waltham, MA, USA). Lysis buffer was purchased from Becton Dickinson (Franklin Lakes, NJ, USA). The glutaraldehyde was from Carl Roth Chemicals (Karlsruhe, Germany) and the absolute ethanol from VWR (Radnor, PA, USA). Human immunoglobulin G1 was obtained from Molecular Innovations (Novi, MI, USA).

### 3.2. Immobilization of Anti-EpCAM onto the Surface of Hollow Fibers

A commercially available Leoceed-16N (Asahi Kasei, Tokyo, Japan) dialyzer was disassembled to obtain the hollow fibers for the parallel immobilization experiments. Fifteen, 13.5 cm long, 215 µm I.D. polysulfone fibers were batched and glued into a same-length polyurethane tube to mimic the original tubes-and-shell arrangement of the dialyzer. The anti-EpCAM antibody was immobilized onto the interior surface (blood side of the dialyzer) of the hemodialysis membrane as follows. The hollow fibers were pretreated with 4% HSA in HPLC grade water using 2 mL/h flow rate for 1 h. Subsequently, 2% glutaraldehyde in HPLC grade water was loaded into the hollow fibers at 0.5 mL/h flow rate for 1 h. Excess of reagents were rinsed out with HPLC grade water at 1 mL/h flow rate for 0.5 h. Then, using a syringe pump (New Era Pump System, Fermingdale, NY, USA), 30 µg/mL anti-EpCAM solution was added to 1 mg/mL picoline borane in 5% EtOH (*V*/*V*) coupling buffer. Control fibers were prepared in the same way, but human IgG1 was used instead of anti-EpCAM. The coupling buffer was run through the system twice at 6 mL/h flow rate for 20 min. Excess reagents were rinsed out with HPLC grade water at 1 mL/h flow rate for 0.5 h. In order to maintain the pH, the hollow fibers were washed with 150 mM of ethanolamine solution at 1 mL/h flow rate for 0.5 h. Finally, the fibers were washed using PBS (pH 7.4) at a 1 mL/h flow rate for 0.5 h. The reported immobilization procedure was based on a systematic parameter optimization (see photo of the experimental setup in the Supplementary Material), including the concentrations of anti-EpCAM, HSA and glutaraldehyde and the contact times of the different solutions. All immobilization steps were carried out at room temperature. The results of the immobilization and capture procedures were examined using a Nikon Eclipse Ni upright fluorescence microscope equipped with a Nikon D5000 digital camera and a DAPI (ex.: 375 nm/em.: 460 nm) bandpass filter cube (Nikon, Tokyo, Japan). For better microscopic visualization, immobilization was accomplished on the outer surface of the hollow fibers. After the capture experiments, the fibers were washed by HPLC grade water, and the anchored cells were stained with the Hoechts 33342 dye (ex.: 350 nm/em.: 461 nm) to minimalize wavelength overlapping with FITC (ex.: 490 nm/em.: 525 nm).

### 3.3. Cell Culturing

Green fluorescent protein (GFP) expressing HCT116 cells were supplied by the National Institute of Oncology (Budapest, Hungary) and cultured in RPMI media containing 11% fetal bovine serum and 1% Penicillin-Streptomycin at 37 ℃ in 5% CO_2_ atmosphere. The colorectal cells were proved to be EpCAM positive [45,46], but EpCAM expression of the utilized cell line was confirmed by FITC labeled anti-EpCAM conjugation as well. The ratio of GFP-expressing and non-expressing cells was measured by flow cytometry and found to be an average of 67%. Cultured cells were detached from the flask walls with cell scrapers before experiments.

### 3.4. Flow Cytometry

A 100 µL cell suspension was taken three times from each media (feed, effluent, etc.). Each sample was injected into the flow cytometer in triplicates. Blood samples were lysed by adding the BD lysing buffer according to the manufacturer’s protocol. To increase the accuracy of the quantitative cell count determination in biological specimens as well as in buffer-cell suspensions, 100 µL of fluorescent microsphere internal standard (Flow Count Fluorospheres, Beckman Coulter, Brea, CA, USA) was added to each sample. In this way, absolute cell count was measured by using a Gallios Flow Cytometer (Beckman Coulter, Brea, CA, USA) enabling forward scatter (FSC) and side scatter (SSC) detectors.

### 3.5. Cell Capture

This study was carried out in accordance with relevant guidelines and regulations, including ARRIVE [47]. Anti-EpCAM immobilized membrane fibers were used during the cell capture experiments. HCT116 cells at ~800 cells/µL concentration were spiked into 0.5 mL PBS buffer and whole blood (mice blood was from National Institute of Oncology (Budapest, Hungary) under the ethical approvals PEI/001-2574-6/2015 and PE/EA/1461-7/2020 issued by Pest County Government Office), collected from the tail vein by syringe and then heparinized according to the standard procedure, i.e., 50 IU/kg body weight [48]). The model solutions were run through the membrane fibers using a precision ultra-low flow peristaltic pump (Ismatech, Wertheim, Germany) at a 6 mL/h flow rate. As non-specific binding of adhesive cells cannot be completely excluded, control experiments were performed to establish a reference for accurate evaluation of the cell capture efficiency. The setup of the control experiments was identical as described above, except human IgG1 was used in the activation step instead of the anti-EpCAM antibody. Furthermore, to get a more accurate insight into the capabilities of the system, model solutions were run in a continuous procedure in a way that the total volume was circulated multiple times. Cell concentrations in the feed and effluent were measured by flow cytometry.

### 3.6. Water Permeability Measurement

Commercially available B. Braun Diacap Lopes low-flux polysulfone dialyzers were used for the permeability tests. A modified dead-end filtration method was applied on both the intact and modified hemo-dialyzers, as suggested by Labib et al. [49]. A total of 13.2 mm Hg (1.76 kPa) pressure was applied on the dialysate-side inlet port. The permeated water was collected from the blood-side exit port, while all other ports were closed. HPLC grade water was used to perform the permeability testing. The volumetric flow rate was measured and used for the calculation of the absolute water permeability.

## 4. Conclusions

In this study, we demonstrated the feasibility of in situ immobilization of human anti-EpCAM antibodies onto the interior surface of commercially available hollow hemodialysis fibers for the selective capture of circulating tumor cells while maintaining its hemodialysis performance. As high as 69% specific capture efficiency was attained from HCT116 cell spiked total blood, quantitated by flow cytometry. The suggested workflow resulted in approximately 2.1 × 10^6^ cells/m^2^ absolute cell capture capacity potential, which is several orders of magnitude higher than what would be required in any patient case. Alternatively to anti-EpCAM, other binding agents can be utilized following the approach described in this paper, e.g., specific antibodies, mucins and/or lectins, oligonucleotides such as aptamers, small binding molecules, ligands and their combinations. In addition to the capture efficiency evaluation, absolute water permeability between the untreated and modified dialyzers was investigated in order to understand any possible effect of the immobilization process on the hemodialysis performance. During this part of the study, only ~7% permeability decline was found, most likely due to the presence of residual proteins in the membrane pores or the alteration in the polymer structure at the molecular level, requiring further investigation. Furthermore, specimen-specific permeability such as dextran clearance was not considered as part of this study as the decrease in water permeability does not represent any adverse effects on the original intended use of the dialyzers, i.e., to remove uremic toxins, inflammatory mediators, and cytokines. More importantly, after the cell capture–hemodialysis treatment, the captured CTCs can be recovered from the dialyzer by trypsinization, opening up the possibility of subsequent molecular pathology investigations.

Based on the reported study and further testing in a clinical environment (with realistic CTC concentration), we envision the development of a new adjuvant cancer therapy in the near future, which can be applied prior, during, and after tumor removal surgery. The modified dialyzer will be attached to an ordinary hemodialysis system that processes patient blood. In this way, CTCs will be captured immediately at a very early appearance. Furthermore, the device will be readily applicable to treat cancer-associated cachexia, which is a major cause of cancer morbidity and mortality.

## Figures and Tables

**Table 1:** Results of the flow cytometry analysis and the calculated capture efficiencies. HCT116 cells were spiked into PBS buffer and whole blood, followed by cell capture experiments utilizing the cell capture technology introduced in this paper. Samples were measured in triplicates. Numbering of the sample ID indicates the corresponding repetitions. The reported cell concentration values were directly measured by flow cytometry utilizing fluorescent microspheres as the internal standard. Absolute cell counts are calculated values.

Initial				Flow Through				Efficiency (%)
Sample ID	Cell Concentration (pcs/µL)	Average Cell Concentration (pcs/µL)	Absolute Cell Count (pcs)	Sample ID	Cell Concentration (pcs/µL)	Average Cell Concentration (pcs/µL)	Absolute Cell Count (pcs)	
PBS-EpCAM 1/1	844	842	420808	PBS-EpCAM 1/1	175	167	83432	80.17
PBS-EpCAM 1/2	848			PBS-EpCAM 1/2	164			
PBS-EpCAM 1/3	832			PBS-EpCAM 1/3	162			
PBS-EpCAM 2/1	771	778	389155	PBS-EpCAM 2/1	112	108	54022	86.12
PBS-EpCAM 2/2	777			PBS-EpCAM 2/2	111			
PBS-EpCAM 2/3	786			PBS-EpCAM 2/3	101			
PBS-EpCAM 3/1	807	795	397403	PBS-EpCAM 3/1	115	109	54593	86.26
PBS-EpCAM 3/2	798			PBS-EpCAM 3/2	103			
PBS-EpCAM 3/3	780			PBS-EpCAM 3/3	109			
PBS-Control 1/1	869	827	413533	PBS-Control 1/1	561	535	267630	35.28
PBS-Control 1/2	811			PBS-Control 1/2	532			
PBS-Control 1/3	801			PBS-Control 1/3	513			
PBS-Control 2/1	700	778	389153	PBS-Control 2/1	483	504	252238	35.18
PBS-Control 2/2	811			PBS-Control 2/2	521			
PBS-Control 2/3	825			PBS-Control 2/3	509			
PBS-Control 3/1	805	818	408873	PBS-Control 3/1	538	533	266692	34.77
PBS-Control 3/2	816			PBS-Control 3/2	537			
PBS-Control 3/3	833			PBS-Control 3/3	525			
Blood-EpCAM 1/1	811	824	412200	Blood-EpCAM 1/1	257	257	128500	68.83
Blood-EpCAM 1/2	812			Blood-EpCAM 1/2	265			
Blood-EpCAM 1/3	850			Blood-EpCAM 1/3	249			
Blood-EpCAM 2/1	791	802	401058	Blood-EpCAM 2/1	211	217	108270	73.00
Blood-EpCAM 2/2	805			Blood-EpCAM 2/2	217			
Blood-EpCAM 2/3	811			Blood-EpCAM 2/3	222			
Blood-EpCAM 3/1	836	840	419902	Blood-EpCAM 3/1	271	269	134253	68.03
Blood-EpCAM 3/2	840			Blood-EpCAM 3/2	266			
Blood-EpCAM 3/3	844			Blood-EpCAM 3/3	269			
Blood-Control 1/1	853	846	423233	Blood-Control 1/1	635	635	317323	25.02
Blood-Control 1/2	841			Blood-Control 1/2	641			
Blood-Control 1/3	846			Blood-Control 1/3	629			
Blood-Control 2/1	813	822	410932	Blood-Control 2/1	666	671	335482	18.36
Blood-Control 2/2	822			Blood-Control 2/2	676			
Blood-Control 2/3	830			Blood-Control 2/3	672			
Blood-Control 3/1	805	808	404175	Blood-Control 3/1	647	645	322735	20.15
Blood-Control 3/2	810			Blood-Control 3/2	650			
Blood-Control 3/3	810			Blood-Control 3/3	640			

## Supplementary Materials

The following are available online, Figure S1: Photo of the experimental setup, which was used for the parameter optimization of the suggested technology.

## Sample Availability

Samples of the compounds are not available from the authors.
