# Supplementary material for: Modification of Hemodialysis Membranes for Efficient Circulating Tumor Cell Capture for Cancer Therapy
Source: Molecules. 2021 Aug 10;26(16):4845. doi: 10.3390/molecules26164845 (PMC8398911; doi:10.3390/molecules26164845)
Supplement: Supplementary file 1 [file molecules-26-04845-s001.zip › molecules-1314089-supplementary.pdf]

Modification of hemodialysis membranes for efficient circulating tumor cell capture for cancer therapy

Gabor Jarvas[1,2], Dora Szerenyi[1], Jozsef Tovari[3], Laszlo Takacs[4], Andras Guttman[1,2,*]

[1]Research Institute of Biomolecular and Chemical Engineering, Faculty of Engineering, University of Pannonia, Veszprem, Hungary

[2]CAPTEC Medical Ltd, Budapest, Hungary

[3]Department of Experimental Pharmacology, National Institute of Oncology, Budapest, Hungary

[4]Laboratory of Monoclonal Antibody Proteomics, Department of Human Genetics, Faculty of Medicine, University of Debrecen, Debrecen, Hungary

*corresponding author: Andras Guttman, e-mail: guttman@mik.uni-pannon.hu

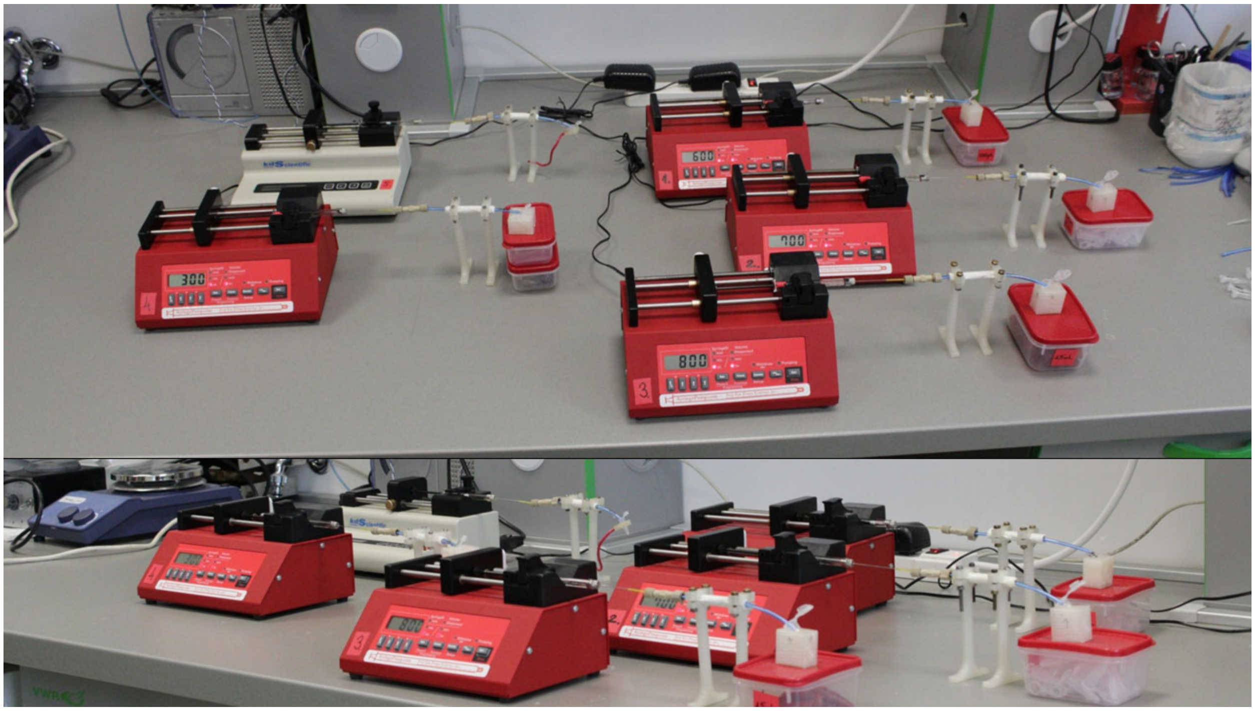

Figure S1 Photo of the experimental setup, which was used for the parameter optimization of the suggested technology.
